# Lumbosacral Transitional Vertebrae Detected on Routine Sacroiliac Joint Radiographs in a Rheumatology Cohort: Comparison of Castellvi and Jenkins Systems

**DOI:** 10.3390/jcm15218436

**Published:** 2026-10-29

**Authors:** Jakub Jankowski, Natalia Maślanek, Marcin Glin, Marcin Jasiewicz, Agnieszka Pawełczyk, Michał Polguj, Maciej Radek

**Affiliations:** 1 Department of Neurosurgery, Spine and Peripheral Nerve Surgery, Medical University of Lodz, Żeromskiego 113, 90-549 Lodz, Poland; natalia.maslanek@stud.umed.lodz.pl (N.M.); marcin.glin@stud.umed.lodz.pl (M.G.); agnieszka.pawelczyk@umed.lodz.pl (A.P.); maciej.radek@umed.lodz.pl (M.R.); 2 Department of Medical Imaging Technology, Faculty of Medicine, Medical University of Lodz, Narutowicza 60, 90-136 Lodz, Poland; marcin.jasiewicz@umed.lodz.pl; 3 Department of Normal and Clinical Anatomy, Anatomy and Histology, Medical University of Lodz, 90-752 Lodz, Poland; michal.polguj@umed.lodz.pl

**Keywords:** Bertolotti syndrome, Castellvi classification, Jenkins classification, low back pain, lumbosacral transitional vertebrae

## Abstract

**Background/Objectives:** Lumbosacral transitional vertebrae (LSTV) are an under-recognized anatomical variant potentially associated with low back pain (LBP). Comparative data on the established Castellvi classification and the more recently proposed Jenkins system are also limited. This study compared LSTV detection by the two systems on routine sacroiliac joint radiographs and evaluated associations with age, LBP, and rheumatologic diagnoses. **Methods:** This retrospective study included 817 patients who underwent sacroiliac joint radiography at a rheumatology outpatient clinic between 2022 and 2024. Radiographs were reassessed using a standardized protocol. Age-related patterns and alternative transverse process–sacral ala distance thresholds were evaluated. Associations between LSTV and documented LBP were adjusted for age, sex, radiographic sacroiliitis grade, and the presence of a recorded rheumatologic diagnosis. **Results:** LSTV was identified in 248/817 patients (30.4%) by Castellvi and in 735/817 (90.0%) by Jenkins. The discrepancy was driven predominantly by Type I variants (20.9% vs. 80.5%); after their exclusion, both systems identified the same 77/817 patients (9.4%) as having Types II–IV. Jenkins-positive prevalence rose from 68.4% in patients younger than 20 years to 100% in those aged ≥70 years (Cochran–Armitage Z = 6.80, *p* < 0.001), whereas no corresponding trend was observed for Castellvi. An exploratory TP–sacral ala threshold of <5 mm reduced Jenkins-positive prevalence to 61.7%. Neither classification was associated with documented LBP after multivariable adjustment. LSTV was not mentioned in any original sacroiliac joint radiology reports. **Conclusions:** The Castellvi and Jenkins systems yielded markedly different LSTV prevalence estimates, primarily because of their differing Type I criteria. The high prevalence and strong age dependence of Jenkins Type I findings suggest that the 10-mm distance criterion may capture age-dependent anatomical configurations in addition to transitional morphology and highlight the need for independent validation before its broader clinical use.

## 1. Introduction

Low back pain (LBP) represents a major diagnostic and therapeutic challenge for specialists in neurosurgery, orthopedics and rheumatology, largely due to the frequent inability to identify a definitive underlying cause. Indeed, the global number of years lived with disability (YLDs) attributable to LBP increased by approximately 54% between 1990 and 2015 [1,2]. Within healthcare systems, the condition constitutes one of the leading causes of multidisciplinary consultations, and its diagnosis and treatment impose a substantial economic burden.

A less common and frequently underrecognized cause of chronic LBP is Bertolotti’s syndrome. This entity is associated with the presence of a lumbosacral transitional vertebra (LSTV), in which the transverse processes (TPs) of the lowest lumbar-type vertebra exhibit a close anatomical relationship with the sacral ala, occasionally forming a pseudoarticulation or osseous connection [3]. LSTV may manifest as sacralization of the lowest lumbar vertebra or lumbarization of the most cranial sacral segment [4,5,6].

The classical Castellvi classification, introduced in 1984, is the most widely used morphological system for classifying LSTV. Type I is defined using a 19-mm craniocaudal threshold for transverse process dysplasia, originally proposed by Southworth and Bersack in 1950 [7]. Types II–IV are defined according to the presence and laterality of pseudoarticulation or osseous fusion between the transverse process and the sacrum. In contrast, the more recent Jenkins classification, proposed in 2023 and derived from clinical and surgical experience [8], uses the TP–sacral ala distance as a central criterion, with a distance of <10 mm defining Type I morphology. This approach was intended to identify anatomical configurations potentially associated with mechanical conflict, even in the absence of pseudoarticulation or fusion. Thus, the two systems represent distinct approaches: Castellvi provides an established morphological framework, whereas Jenkins incorporates a proximity-based criterion intended to capture potentially clinically relevant configurations. However, the historical 19-mm Castellvi threshold has not been comprehensively validated using contemporary morphometric imaging, while the clinical and anatomical significance of the 10-mm Jenkins threshold remains uncertain and has not yet been independently validated against an anatomical reference standard.

The primary objective of the present study was to directly compare the Castellvi and Jenkins classification systems in a large rheumatology cohort undergoing routine sacroiliac joint radiography, with particular emphasis on differences in the criteria used to define Type I variants and their impact on LSTV prevalence and subtype distribution. This cohort was selected because it provided standardized imaging of the lumbosacral junction together with detailed clinical data on LBP and rheumatologic diagnoses. Secondary objectives included assessment of age-related prevalence patterns, the influence of alternative TP–sacral ala distance thresholds, associations with LBP and rheumatologic diagnoses, interobserver reproducibility, and recognition of LSTV in routine radiological reports.

## 2. Materials and Methods

The study dataset was derived from a retrospective review of outpatient medical records and radiographic examinations of patients evaluated at a rheumatology outpatient clinic at a single hospital in Poland between 2022 and 2024. The project was approved by the Bioethics Committee of the Medical University of Lodz RNN/09/25/KE. All procedures were performed in accordance with relevant guidelines and regulations. Given the retrospective design and the use of fully anonymized imaging data, no individual informed consent was required from the participants.

This retrospective observational study was reported in accordance with the Strengthening the Reporting of Observational Studies in Epidemiology (STROBE) guidelines.

To be included in the study, the participants had to be registered in the rheumatology outpatient clinic during the specified period and have been referred for sacroiliac joint radiography during diagnostic workup. The study was conducted in an adult rheumatology outpatient setting; therefore, all screened and included patients were aged 18 years or older.

The resulting patient database was organized using Microsoft Excel. All patients referred for sacroiliac joint radiography during the study period (*N* = 1257) were initially considered for inclusion. The following exclusion criteria were applied:

Failure to undergo sacroiliac joint radiography despite a formal referral (*n* = 397);

Duplicate records pertaining to the same patient (*n* = 24);

Insufficient imaging coverage preventing adequate visualization of the transverse processes of the last presacral vertebra (*n* = 17);

A history of spinal surgery involving instrumentation at the L5–S1 level, precluding reliable radiological assessment (*n* = 2).

**Figure 1:**
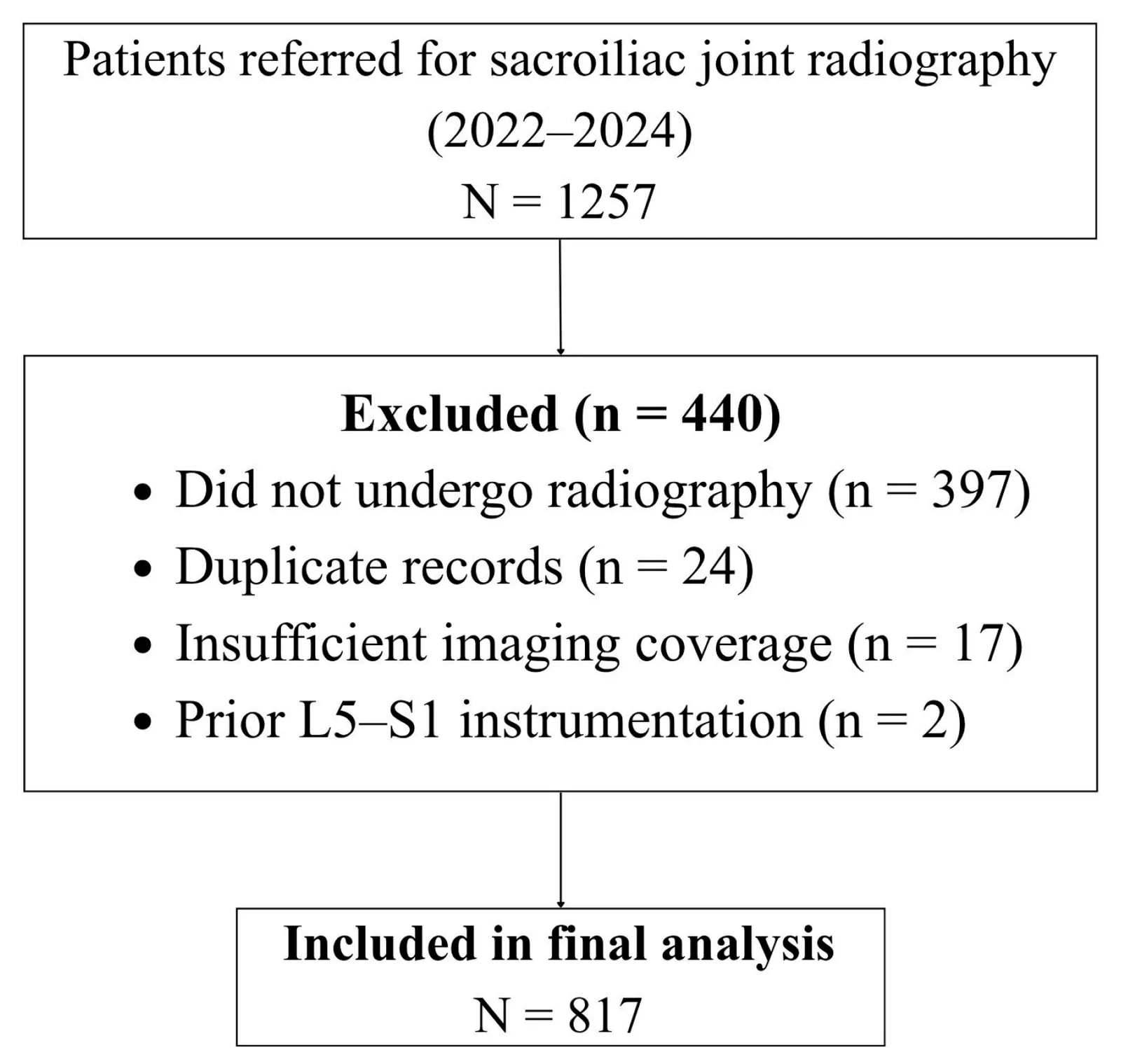
Flow diagram of patient selection.

After excluding *n* = 440 cases, the remaining *N* = 817 participants were included in the final analysis. The patient selection process is summarized in Figure 1. The initial classification was established by consensus between a spine neurosurgeon and an intern physician. All 817 radiographs were then independently re-evaluated by an additional neurosurgeon who was blinded to the consensus classification.

The sacroiliac joints were imaged using two digital radiography systems: Ysio X.pree (Siemens Healthineers) and DigitalDiagnost C90 (Philips Healthcare). All examinations followed a standardized institutional protocol to ensure reproducibility. Patients were positioned supine, and standard anteroposterior projections were obtained with the central beam directed to the midline at the level of the sacrum. Beam angulation and patient positioning were adjusted as needed to optimize visualization of the joint spaces and minimize superimposition.

Automatic exposure control was used in all examinations. Tube voltage was selected according to patient body habitus within the diagnostic range for pelvic imaging. Images were acquired with digital flat-panel detectors, processed using manufacturer-specific algorithms, and transferred to the PACS system. Radiation dose was optimized according to the ALARA principle.

Image analysis was performed using RadiAnt DICOM Viewer version 2025.2 (Medixant, Poznań, Poland). The radiological reports accompanying the analyzed sacroiliac joint radiographs were systematically reviewed for any mention of LSTV to assess its recognition in routine radiological practice. Patient characteristics, imaging findings, rheumatologic diagnoses, and report-review data were systematically entered into a structured database. Microsoft Excel (Microsoft Corporation, Redmond, WA, USA) was used for database organization, data cleaning, and verification. Statistical analyses were performed using TIBCO Statistica version 13.3 (TIBCO Software Inc., Palo Alto, CA, USA) and Python version 3.14.3 (Python Software Foundation), with the NumPy, pandas, SciPy, and statsmodels packages. Logistic regression models and the Benjamini–Hochberg procedure were implemented using statsmodels, while the Cochran–Armitage trend tests were calculated in Python using NumPy and SciPy. All patient data were de-identified before analysis. Radiographic images selected for presentation were fully anonymized to prevent patient identification.

Each case underwent an individualized radiological assessment of the sacroiliac joint radiographs, including a detailed evaluation of the lumbosacral junction. For the purposes of the study, the most caudal presacral vertebra with lumbar-type morphology visible on the radiograph was used as the index vertebra for classification. All images were analyzed according to a predefined, standardized five-step protocol.

First, the visibility of the most caudal transverse processes was assessed using image magnification (150–200%) and optimized windowing.

Second, the morphology of the transverse processes was evaluated to distinguish true pseudoarticulation from simple bony contact. A true pseudoarticulation was identified by the presence of a distinct horizontal radiolucent cleft between the transverse process and the sacral ala, typically accompanied by remodeling of the adjacent sacral contour. In contrast, simple bony contact was defined as close apposition or apparent touching without a distinct horizontal radiolucent cleft or characteristic remodeling of the sacral contour. Cases with a measured distance < 1 mm that lacked these morphological features of pseudoarticulation were classified as Type I (proximity/contact) rather than Type II.

Third, the maximum craniocaudal height of the transverse processes was measured.

Fourth, the shortest distance between the inferior cortex of the transverse process and the superior cortex of the sacral ala was measured. When the transverse process was markedly inclined, or the alar contour was poorly defined, the measurement was taken at the point of closest approximation; if the contour remained insufficiently visible, the side was recorded as not assessable. No correction for radiographic magnification was applied. Measurements were performed directly on the PACS images obtained using the standardized institutional radiographic protocol.

Fifth, each case was classified according to both the Castellvi and Jenkins systems.

For the interobserver reliability analysis, the independent reassessment of the entire cohort of 817 radiographs was compared with the consensus classification recorded in the final study database. The additional neurosurgeon independently assessed each radiograph and assigned Castellvi and Jenkins classifications according to the predefined study criteria, while remaining blinded to the consensus classification. Agreement for exact subtype classification according to the Castellvi and Jenkins systems was assessed using unweighted Cohen’s kappa with 95% confidence intervals. Agreement for the binary classification of LSTV as present or absent was assessed separately using unweighted Cohen’s kappa. Because of the high prevalence of Jenkins-positive findings, prevalence-adjusted measures of agreement, including the prevalence-adjusted bias-adjusted kappa (PABAK) and Gwet’s AC1, were additionally calculated. Exact percentage agreement was also reported.

In addition, patient-specific clinical and demographic data were systematically collected. These data included patient sex, age, nationality, and radiographic grading of sacroiliitis according to the modified New York criteria. Each sacroiliac joint was graded separately from 0 to 4; for patient-level analyses, the highest grade observed in either joint was used. A structured review of the available medical records was also performed, with particular emphasis on the presence of LBP and recorded rheumatologic diagnoses. Rheumatologic diagnoses documented up to February 2026 were included. Diagnostic categories were not mutually exclusive because some patients had more than one rheumatologic diagnosis.

LBP was defined as documented pain localized to the lower back. The retrospective medical records did not permit consistent classification of pain according to duration, mechanical versus inflammatory characteristics, laterality, or attribution to the sacroiliac joint or transitional lumbosacral anatomy. Accordingly, the study evaluated the association between radiographic LSTV morphology and documented LBP but did not establish or assess a diagnosis of Bertolotti syndrome.

Continuous variables were summarized using means with standard deviations and medians with ranges, as appropriate, whereas categorical variables were reported as counts and percentages. Paired differences in LSTV detection between the Castellvi and Jenkins systems were evaluated using McNemar’s test. Associations between categorical variables were assessed using Pearson’s χ^2^ test or Fisher’s exact test when expected cell counts were small. Linear trends across ordered age and radiographic sacroiliitis categories were evaluated using the Cochran–Armitage trend test. In additional analyses, age was evaluated as a continuous variable using sex-adjusted logistic regression models. Associations were expressed as adjusted odds ratios (aORs) with 95% confidence intervals (CIs) per 10-year increase in age. Outcomes included overall Jenkins-positive status, overall Castellvi-positive status, Jenkins Type I, Jenkins Types II–IV, TP–sacral ala distance < 5 mm, and bony contact < 1 mm. Effect sizes for categorical associations were expressed using Cramér’s V where appropriate. ORs with 95% CIs were calculated for binary outcomes. Associations of Castellvi-positive status, Jenkins-positive status, and Types II–IV with LBP were additionally evaluated using separate multivariable logistic regression models adjusted for age, sex, radiographic sacroiliitis grade (entered as a categorical variable), and the presence of a recorded rheumatologic diagnosis. Logistic regression models adjusted for age and sex were used for prespecified disease-specific analyses of LSTV and LBP, with patients without a recorded rheumatologic diagnosis serving as the reference group. The Benjamini–Hochberg false-discovery-rate procedure was applied separately within each disease-specific outcome across the seven major diagnostic categories (AS, PsA, degenerative joint disease, nr-axSpA, RA, SLE, and UCTD). Where complete separation precluded stable estimation of an adjusted odds ratio, an age- and sex-adjusted score test was used for hypothesis testing, and the resulting *p* value was included in the Benjamini–Hochberg correction. This score test was implemented using statsmodels and SciPy. All tests were two-sided, and *p* < 0.05 was considered statistically significant. Post hoc analyses using TP–sacral ala thresholds of <5 mm and <1 mm were considered exploratory.

## 3. Results

A total of 817 patients were ultimately included in the detailed radiographic analysis of the sacroiliac joints: 529 women (64.7%) and 288 men (35.3%). The mean age was 48.3 ± 14.7 years, with a median of 48 years and a range of 18–87 years. The cohort was predominantly Polish, with 811/817 patients (99.3%) recorded as having Polish nationality; 6 patients (0.7%) had other nationalities.

### 3.1. Interobserver Reliability

Interobserver agreement was assessed by independent re-evaluation of all 817 cases in the study cohort. Exact subtype agreement was observed in 671/817 cases (82.1%) for the Castellvi classification and in 703/817 cases (86.0%) for the Jenkins classification. Unweighted Cohen’s kappa for exact subtype classification was 0.656 (95% CI 0.608–0.705) for Castellvi and 0.707 (95% CI 0.661–0.753) for Jenkins.

For the binary classification of LSTV as present or absent, exact agreement was 712/817 (87.1%) for Castellvi and 779/817 (95.3%) for Jenkins. Cohen’s kappa was 0.710 (95% CI 0.658–0.761) for Castellvi and 0.725 (95% CI 0.641–0.808) for Jenkins. Given the high prevalence of Jenkins-positive findings, prevalence-adjusted agreement coefficients were additionally calculated for both classifications. PABAK was 0.743 for Castellvi and 0.907 for Jenkins, while Gwet’s AC1 was 0.770 and 0.944, respectively. Most disagreements involved Type I variants, particularly distinctions between negative findings and Type I variants, as well as between Type IA and Type IB configurations.

The independent observer assessed the final Castellvi and Jenkins classifications but did not independently repeat the TP–sacral ala distance measurements.

### 3.2. Prevalence According to the Castellvi and Jenkins Classifications

**Figure 2:**
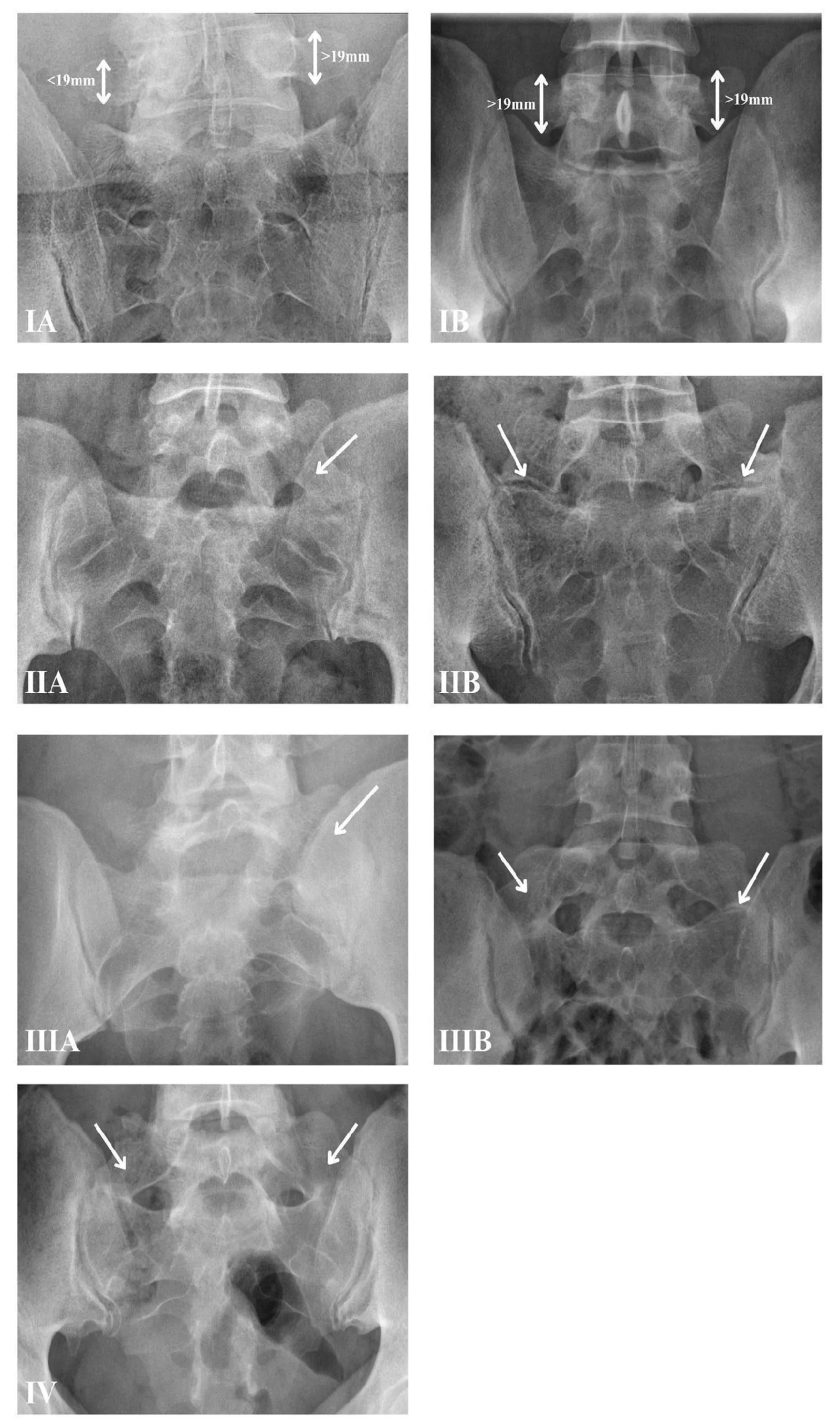
Representative anteroposterior radiographs demonstrating the Castellvi classification of LSTV. Type (IA)—unilateral enlarged TP measuring ≥ 19 mm; contralateral side normal. Type (IB)—bilateral enlarged TP measuring ≥ 19 mm bilaterally. Type (IIA)—unilateral pseudoarticulation between the enlarged TP and the sacral ala; contralateral side normal. Type (IIB)—bilateral pseudoarticulations between the TP and the sacral ala. Type (IIIA)—unilateral complete osseous fusion of the TP with the sacrum; contralateral side normal. Type (IIIB)—bilateral complete osseous fusion of the TP with the sacrum. Type (IV)—mixed morphology with pseudoarticulation on one side and complete osseous fusion on the contralateral side.

Assessment of the lumbosacral junction on sacroiliac joint radiographs revealed the presence of LSTV in 248/817 individuals (30.4%) according to the Castellvi classification (Figure 2). Among these, the predominant variant was Type IA, identified in 94/248 cases (37.9%). This was followed by Type IB, in 77/248 cases (31.0%), Type IIA in 29/248 cases (11.7%), Type IIB in 17/248 cases (6.9%), Type IIIA in 12/248 cases (4.8%), Type IIIB in 12/248 cases (4.8%), and Type IV in 7/248 cases (2.8%).

**Figure 3:**
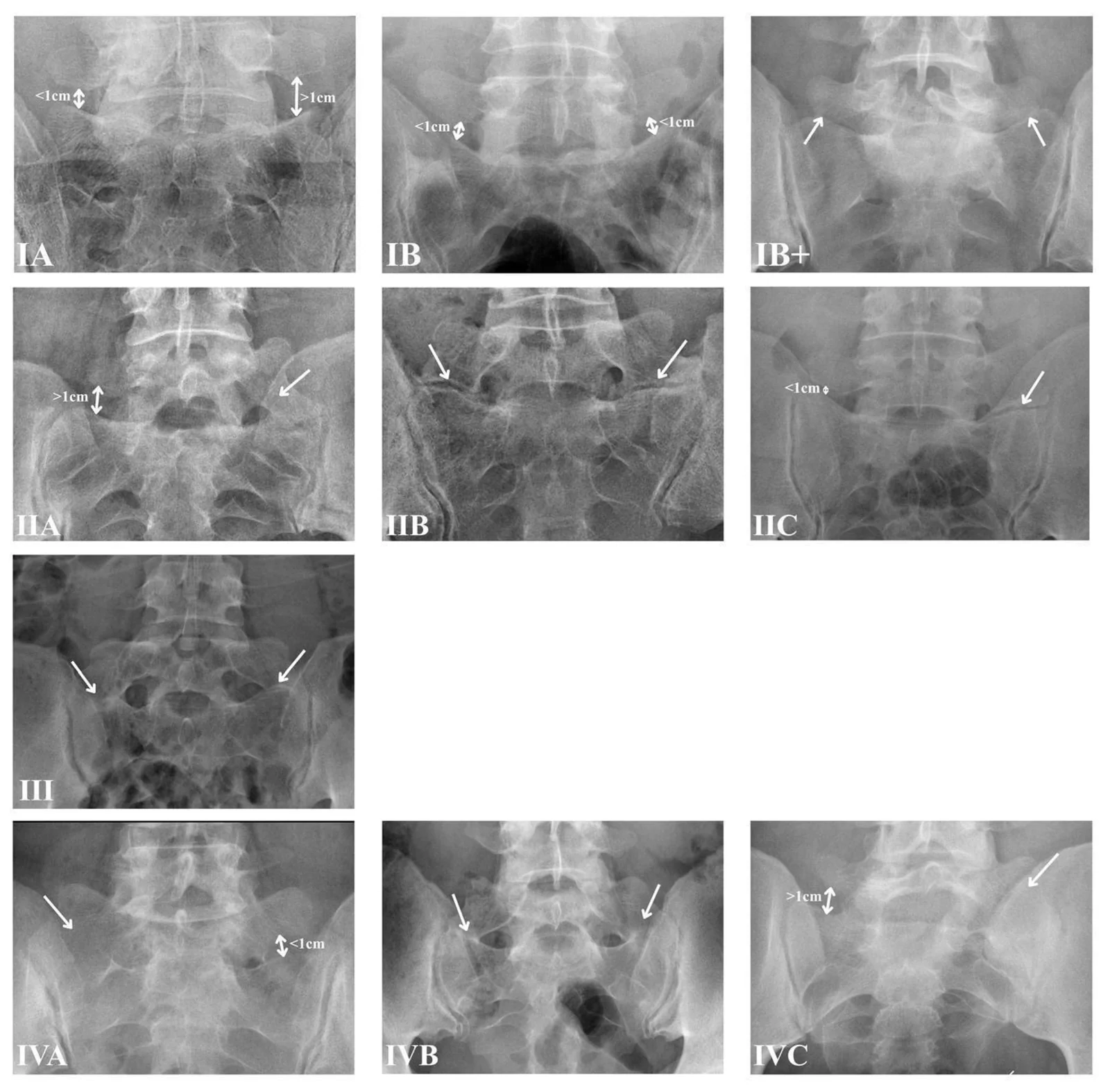
Representative anteroposterior radiographs demonstrating the Jenkins classification of LSTV. Type (IA)—TP–sacral ala distance < 10 mm; contralateral side normal (>10 mm). Type (IB)—TP–sacral ala distance < 10 mm bilaterally. Type (IB+)—an illustrative near-contact variant within Type (IB) (TP–sacral ala distance < 2 mm bilaterally), not an additional Jenkins subtype. Type (IIA)—unilateral pseudoarticulation between the TP and the sacral ala; contralateral side normal. Type (IIB)—bilateral pseudoarticulations between the TP and the sacral ala. Type (IIC)—mixed morphology with pseudoarticulation on one side and Type I morphology on the contralateral side. Type (III)—bilateral complete osseous fusion of the TP with the sacrum. Type (IVA)—unilateral osseous fusion of the TP with the sacrum; contralateral side demonstrating Type I morphology. Type (IVB)—unilateral osseous fusion of the TP with the sacrum; contralateral side demonstrating pseudoarticulation. Type (IVC)—unilateral osseous fusion of the TP with the sacrum; contralateral side normal.

In contrast, the same cohort yielded a markedly higher overall prevalence of LSTV based on the Jenkins classification (Figure 3), with positive findings identified in 735/817 individuals (90.0%). The most common subtype under this classification was Type IB, present in 575/735 cases (78.2%). This was followed by Type IA in 83/735 cases (11.3%), Type IIC in 20/735 cases (2.7%), Type IIB in 17/735 cases (2.3%), Type III in 12/735 cases (1.6%), Type IIA in 9/735 cases (1.2%), Type IVB in 7/735 cases (1.0%), Type IVA in 6/735 cases (0.8%), and Type IVC in 6/735 cases (0.8%). Types IVA and IVC were the least frequent subtypes.

Of the 817 patients, 246 were classified as LSTV-positive by both systems, 489 were positive only according to Jenkins, two were positive only according to Castellvi, and 80 were negative according to both systems. The difference in detection rates was significant in the paired analysis (McNemar’s test, *p* < 0.001).

The marked difference between the classifications was predominantly attributable to Type I variants. Castellvi Type I was identified in 171/817 patients (20.9%), whereas Jenkins Type I was identified in 658/817 patients (80.5%). After Type I variants were excluded, the prevalence was identical for the two classification systems: Types II–IV were identified in 77/817 patients (9.4%) according to both Castellvi and Jenkins. The same 77 patients were included in this category by both systems.

Cross-tabulation of individual subtypes further demonstrated the extent of this discrepancy. Among 569 patients classified as negative according to Castellvi, 489 were classified as Jenkins-positive, including 69 as Type IA and 420 as Type IB. Of the 171 patients with Castellvi Type I morphology, 169 were Jenkins-positive, whereas two Castellvi Type IB cases were Jenkins-negative. The complete subtype cross-tabulation is presented in Supplementary Table S2.

### 3.3. Sensitivity Analyses of the Distance Criterion

To assess the influence of the distance criterion on Jenkins Type I classification, a post hoc sensitivity analysis was performed using a more restrictive TP–sacral ala distance threshold of <5 mm. Under this modified criterion, 427 patients met the criterion for Type I, including 184 unilateral and 243 bilateral cases. Together with the 77 patients classified as Jenkins Types II–IV, this resulted in a modified overall Jenkins-positive prevalence of 504/817 (61.7%). The number of Type IB cases decreased by 332 (57.7%), from 575 to 243, whereas the number of Type IA cases increased by 101, from 83 to 184. This reflected both the reclassification of some previously bilateral Type IB variants as unilateral Type IA and the exclusion of cases that no longer met the more restrictive distance criterion.

Among cases originally classified as Jenkins Type I, radiographic near-contact, operationally defined as a TP–sacral ala distance of <1 mm, was observed in 103/658 (15.7%) patients. Of these, only two were Type IA (2/83; 2.4%), while the remaining 101 were Type IB (101/575; 17.6%). Notably, in 89 of the 101 Type IB cases with bony contact (88.1%), the contralateral side also showed a TP–sacral ala distance of <5 mm.

In an additional exploratory analysis, if a distance of <1 mm were applied as a highly restrictive proximity threshold for Jenkins Type I, together with the higher-grade variants (Types II–IV), the overall prevalence of Jenkins-positive cases would decrease to 180/817 cases (22.0%).

### 3.4. Relationship Between Castellvi Type I Morphology and the Distance Criterion

Regarding the association between Castellvi Type I morphology and the Jenkins distance criterion, 75/94 Castellvi Type IA cases (79.8%) demonstrated a TP–sacral ala distance of <5 mm. Among these 75 cases, the reduced distance occurred on the same side as the enlarged transverse process, or the distance reduction was bilateral, in 67 (89.3%). Similarly, 61/77 Castellvi Type IB cases (79.2%) demonstrated a TP–sacral ala distance of <5 mm on at least one side, with bilateral distance reduction observed in 50/61 cases (82.0%). These findings indicate that the presence of transverse process dysplasia according to the Castellvi classification was significantly associated with a reduced TP–sacral ala distance of <5 mm (χ^2^ = 64.45, df = 1, *p* < 0.001; Cramér’s V = 0.281).

However, the reverse relationship does not hold to the same extent: a reduced distance of <5 mm was frequently observed in cases lacking transverse process dysplasia. Of the 427 cases with a distance below 5 mm, only 136 (31.9%) fulfilled the morphological criteria for Castellvi Type I, indicating that a short TP–sacral distance alone does not imply the presence of an enlarged or dysplastic transverse process.

Interestingly, radiographic near-contact (distance < 1 mm) was observed in 45/171 patients (26.3%) with Castellvi Type I morphology, including 19/94 Type IA cases (20.2%) and 26/77 Type IB cases (33.8%). Overall, 45/103 patients (43.7%) meeting the <1-mm criterion had Castellvi Type I morphology, whereas 58/103 (56.3%) did not.

**Figure 4:**
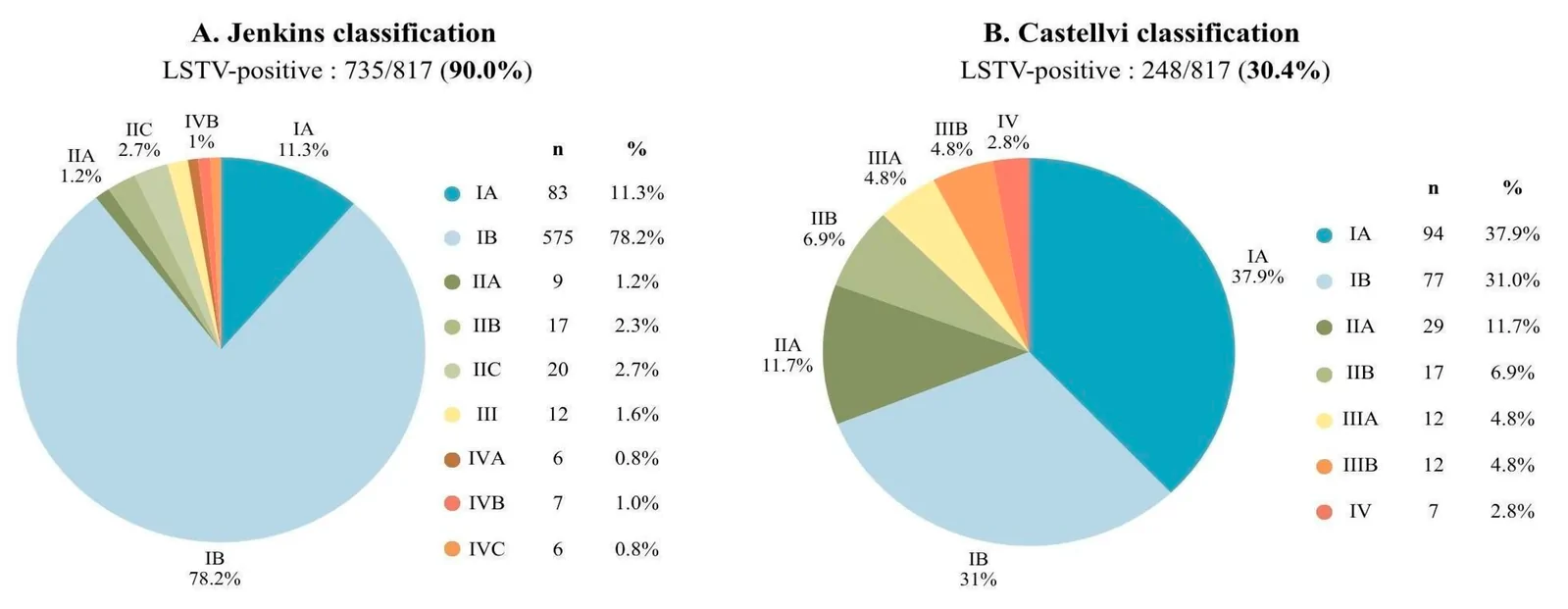
LSTV subtypes according to the Jenkins (**A**) and Castellvi (**B**) classification systems. Values represent the number of cases and the proportion of each subtype among all LSTV-positive patients classified using the respective system. The Jenkins classification was dominated by Type IB (78.2%), whereas Castellvi Type I variants (Types IA and IB combined) accounted for 69.0% of all Castellvi-positive cases.

The relative distributions of individual LSTV subtypes according to the Castellvi and Jenkins classifications are compared in Figure 4.

### 3.5. Age-Related Prevalence Patterns

Across successive age categories, the proportion of LSTV identified using the Jenkins classification increased progressively. Jenkins-positive findings were observed in 13/19 patients (68.4%) younger than 20 years, 59/80 (73.8%) aged 20–29 years, 113/132 (85.6%) aged 30–39 years, 186/205 (90.7%) aged 40–49 years, 175/188 (93.1%) aged 50–59 years, and 121/125 (96.8%) aged 60–69 years. All patients aged 70–79 years (62/62) and 80–89 years (6/6) were classified as Jenkins-positive. The Cochran–Armitage trend test demonstrated a significant positive trend in Jenkins-positive prevalence across increasing age categories (Z = 6.80, two-sided *p* < 0.001).

By comparison, the frequency of Castellvi-positive findings showed minimal variation between age groups (36.8%, 28.8%, 29.5%, 29.8%, 31.9%, 32.8%, 24.2%, and 33.3%, respectively), with no evidence of a significant linear trend (Z = −0.09, *p* = 0.929). These findings are shown in Table 1.

The age-related increase in Jenkins-positive findings was concentrated in Type I variants (Z = 5.64, *p* < 0.001), whereas no significant trend was observed for Jenkins Types II–IV (Z = −0.65, *p* = 0.516). Similarly, no significant age-related trends were observed for Castellvi Type I (Z = 0.37, *p* = 0.714) or Castellvi Types II–IV (Z = −0.65, *p* = 0.516). The prevalence of a TP–sacral ala distance of <5 mm (Z = 7.14, *p* < 0.001) and <1 mm (Z = 6.60, *p* < 0.001) also increased significantly with age. In complementary analyses treating age as a continuous variable, each 10-year increase in age was associated with higher odds of Jenkins-positive classification after adjustment for sex (aOR = 1.86, 95% CI 1.55–2.24; *p* < 0.001). Similar associations were observed for Jenkins Type I (aOR = 1.44, 95% CI 1.27–1.63; *p* < 0.001), TP–sacral ala distance < 5 mm (aOR = 1.44, 95% CI 1.30–1.60; *p* < 0.001), and bony contact < 1 mm (aOR = 1.67, 95% CI 1.43–1.95; *p* < 0.001). In contrast, age was not associated with Castellvi-positive status (aOR = 1.00, 95% CI 0.90–1.11; *p* = 0.970) or Jenkins Types II–IV (aOR = 0.96, 95% CI 0.81–1.12; *p* = 0.584).

### 3.6. Recognition in Routine Radiological Reports

None of the original sacroiliac joint radiology reports explicitly mentioned LSTV. Among the 817 patients studied between 2022 and 2024, LSTV were identified in 248 patients according to the Castellvi classification and in 735 patients according to the Jenkins classification, but none of these findings were documented in the original sacroiliac joint radiology reports.

In all included examinations, the transverse processes of the lowest lumbar-type vertebra and their relationship with the sacral alae were considered assessable according to the predefined study criteria. Radiological reports were limited to abnormalities of the sacroiliac joints, such as subchondral sclerosis assessed according to the modified New York criteria, without reference to coexisting lumbosacral anatomical variants.

### 3.7. Radiographic Sacroiliitis

Radiographic evaluation of the sacroiliac joints was performed according to the modified New York criteria. Based on the highest grade observed in either sacroiliac joint, Grade 0 was identified in 210/817 patients (25.7%), Grade 1 in 313/817 (38.3%), Grade 2 in 228/817 (27.9%), Grade 3 in 53/817 (6.5%), and Grade 4 in 13/817 (1.6%).

The degree of involvement differed between the right and left sacroiliac joints in 228/817 patients (27.9%). Asymmetric involvement was defined as a unilateral abnormality or unequal grades between the right and left sacroiliac joints. A detailed distribution of all side-specific configurations and coexisting LSTV findings is presented in Supplementary Table S1.

The prevalence of LSTV did not differ significantly between radiographic sacroiliitis grades according to either the Castellvi classification (χ^2^ = 4.13, df = 4, *p* = 0.389) or the Jenkins classification (χ^2^ = 0.49, df = 4, *p* = 0.975). No significant association was observed after Type I variants were excluded. As the same 77 patients were classified as Types II–IV by both systems, the association between Types II–IV and radiographic sacroiliitis grade was identical for the Castellvi and Jenkins classifications (χ^2^ = 2.13, df = 4, *p* = 0.712).

A significant association was observed between higher radiographic grade and LBP according to the modified New York criteria (χ^2^ = 25.60, df = 4, *p* < 0.001). The prevalence of LBP increased across successive radiographic categories, from 119/210 (56.7%) in Grade 0 to 180/313 (57.5%) in Grade 1, 164/228 (71.9%) in Grade 2, 43/53 (81.1%) in Grade 3 and 11/13 (84.6%) in Grade 4. A significant positive linear trend was confirmed (Z = 4.66, *p* < 0.001; Cochran–Armitage test), indicating a higher prevalence of LBP with increasing radiographic severity.

### 3.8. Rheumatologic Diagnoses

Of the 817 patients, 450 (55.1%) had at least one recorded rheumatologic diagnosis by February 2026. The remaining 367 patients (44.9%) had not been assigned a definitive diagnosis, either because of the absence of a rheumatic condition, discontinuation of the diagnostic process, or failure to meet established classification criteria.

Among the 450 patients with a recorded rheumatologic diagnosis, the most prevalent conditions were ankylosing spondylitis (AS), identified in 128/450 patients (28.4%), and psoriatic arthritis (PsA), identified in 116/450 (25.8%). These were followed by degenerative joint disease in 61/450 patients (13.6%), non-radiographic axial spondyloarthritis (nr-axSpA) in 52/450 (11.6%), rheumatoid arthritis (RA) in 52/450 (11.6%), systemic lupus erythematosus (SLE) in 13/450 (2.9%), and undifferentiated connective tissue disease (UCTD) in 10/450 (2.2%). At least one other less frequent diagnosis was recorded in 38/450 patients (8.4%). This category included fibromyalgia, diffuse idiopathic skeletal hyperostosis (DISH), juvenile idiopathic arthritis, Sjögren’s syndrome, polymyalgia rheumatica, inflammatory myopathy, inflammatory bowel disease-associated spondyloarthritis, reactive arthritis, mixed connective tissue disease, gout, SAPHO syndrome, Lyme disease, Ormond disease, and chronic post-infectious arthropathy.

As some patients had multiple concomitant rheumatologic diagnoses, the cumulative number of diagnoses exceeded the number of patients with a recorded diagnosis. Multiple diagnoses were recorded in 20/450 patients (4.4%): nineteen patients had two diagnoses and one patient had three. The “Other diagnoses” category comprised 38 patients but 39 individual diagnoses because one patient had two diagnoses assigned to this category. Consequently, the eight category-level patient counts sum to 470, whereas 471 individual diagnoses were recorded. The most frequent overlaps were AS with RA (*n* = 3), degenerative joint disease with DISH (*n* = 3), AS with degenerative joint disease (*n* = 2), and PsA with RA (*n* = 2). The only triple-diagnosis combination occurred in a single patient with concomitant AS, SLE, and Lyme disease.

### 3.9. Associations Between LSTV and Rheumatologic Diagnoses

The presence of a recorded rheumatologic diagnosis was not associated with overall LSTV prevalence according to either classification system. Castellvi-positive findings were identified in 140/450 patients with a recorded diagnosis and in 108/367 patients without a recorded rheumatologic diagnosis (31.1% vs. 29.4%; OR = 1.08, 95% CI 0.80–1.46; *p* = 0.603). Similarly, Jenkins-positive findings were identified in 404/450 patients with a diagnosis and 331/367 without (89.8% vs. 90.2%; OR = 0.96, 95% CI 0.60–1.51; *p* = 0.845).

After the Type I variants were excluded, Types II–IV were identified in 49/450 patients with a diagnosis and in 28/367 patients without (10.9% vs. 7.6%; OR = 1.48, 95% CI 0.91–2.41; *p* = 0.113). As the same 77 patients were classified as Types II–IV by both systems, the result was identical for the Castellvi and Jenkins classifications.

Age- and sex-adjusted disease-specific analyses were performed for all seven prespecified diagnostic categories, using patients without a recorded rheumatologic diagnosis as the reference group. No diagnostic category was significantly associated with either Castellvi-positive or Jenkins-positive status after Benjamini–Hochberg correction. For Types II–IV, RA showed a nominal association, with these variants identified in 10/52 patients (19.2%; aOR = 3.01, 95% CI 1.32–6.90; raw *p* = 0.009); however, this association did not remain statistically significant after correction for multiple comparisons (BH-adjusted *p* = 0.063). No other disease-specific association with Types II–IV was statistically significant after correction. Complete crude counts, adjusted odds ratios with 95% confidence intervals, raw *p* values, and BH-adjusted *p* values for all seven diagnostic categories are presented in Table 2.

### 3.10. Associations with Low Back Pain

LBP was documented in 517/817 patients (63.3%). The prevalence did not differ between Castellvi-positive and Castellvi-negative patients (158/248, 63.7% vs. 359/569, 63.1%; *p* = 0.867) or between Jenkins-positive and Jenkins-negative patients (466/735, 63.4% vs. 51/82, 62.2%; *p* = 0.830). In multivariable logistic regression models adjusted for age, sex, radiographic sacroiliitis grade, and the presence of a recorded rheumatologic diagnosis, neither Castellvi-positive status (aOR = 1.04, 95% CI 0.75–1.44; *p* = 0.819) nor Jenkins-positive status (aOR = 1.41, 95% CI 0.85–2.33; *p* = 0.184) was associated with LBP. LBP was present in 43/77 patients with Types II–IV (55.8%), compared with 474/740 patients without these variants (64.1%; *p* = 0.155). This association remained non-significant in the multivariable model (aOR = 0.66, 95% CI 0.41–1.08; *p* = 0.101). Because the same 77 patients were classified as Types II–IV by both systems, the result was identical for the Castellvi and Jenkins classifications. LBP was documented in 306/450 patients with a recorded rheumatologic diagnosis (68.0%) and in 211/367 patients without a recorded rheumatologic diagnosis (57.5%). The presence of LBP was significantly associated with a recorded rheumatologic diagnosis (χ^2^ = 9.60, df = 1, *p* = 0.002). Patients with a recorded rheumatologic diagnosis had higher odds of LBP than those without a definitive diagnosis (OR = 1.57, 95% CI 1.18–2.09).

Using patients without a recorded rheumatologic diagnosis as the reference group, age- and sex-adjusted disease-specific analyses showed significantly higher odds of LBP in patients with AS (110/128, 85.9%; aOR = 5.54, 95% CI 3.15–9.76; raw *p* < 0.001; BH-adjusted *p* < 0.001), degenerative joint disease (44/61, 72.1%; aOR = 2.63, 95% CI 1.39–4.96; raw *p* = 0.003; BH-adjusted *p* = 0.007), and nr-axSpA (41/52, 78.8%; aOR = 3.37, 95% CI 1.65–6.91; raw *p* < 0.001; BH-adjusted *p* = 0.003). No statistically significant associations with LBP were observed for PsA, RA, SLE, or UCTD after correction for multiple comparisons.

LBP was documented in 211/367 patients without a recorded rheumatologic diagnosis (57.5%). In this subgroup, Castellvi-positive findings were identified in 108 patients, of whom 60 had documented LBP. The prevalence of LBP did not differ between Castellvi-positive and Castellvi-negative patients (60/108, 55.6% vs. 151/259, 58.3%; OR = 0.89, 95% CI 0.57–1.41; *p* = 0.628). This lack of association persisted after adjustment for age, sex, and radiographic sacroiliitis grade (aOR = 1.07, 95% CI 0.66–1.73; *p* = 0.791). LBP was documented in 47/80 patients with Castellvi Type I (58.8%), 5/13 with Type II (38.5%), 7/13 with Type III (53.8%), and 1/2 with Type IV (50.0%). Due to the small numbers of patients with Castellvi Types II–IV, particularly Type IV (*n* = 2), the subtype-specific findings were presented descriptively, and no formal statistical comparison between individual Castellvi types was performed.

## 4. Discussion

This study directly compared the Castellvi and Jenkins classification systems in a large rheumatology cohort and demonstrated markedly different prevalence estimates, driven predominantly by their differing Type I criteria. Although both systems are used to describe LSTV, they differ substantially in their underlying criteria and have important limitations. Neither system distinguishes lumbarization from sacralization; rather, both describe the morphology or spatial relationship of the transverse processes without providing a comprehensive assessment of the lumbosacral junction. Recent studies have attempted a more holistic morphological evaluation of this region. Krenn et al. demonstrated that sacral segmentation anomalies are frequent (approximately 40% prevalence) and produce predictable morphological patterns that can help differentiate lumbarization from sacralization [9]. Similarly, Wu et al. proposed a rapid, practical method for identifying lumbarization on standard lumbar radiographs and CT based on specific sacral and L5 morphological features [10].

The Castellvi classification incorporates a 19-mm radiographic threshold for transverse process dysplasia [7]. This threshold was originally proposed in 1950 and has not been comprehensively validated using modern CT-based morphometric methods, despite the transverse process being a three-dimensional, inclined, and rotated structure. Whether it remains an appropriate criterion for defining Type I variants therefore requires further anatomical validation. Moreover, the Castellvi system may not identify cases of complete lumbarization (six lumbar vertebrae with a fully separated sacral body) in which the transverse processes are morphologically normal.

The Jenkins classification introduces a potentially clinically relevant distance-based criterion. However, the 10-mm threshold was derived from the experience of a single centre and has not undergone independent scientific validation [8]. In the present cohort, most patients with Castellvi Type I morphology also showed a reduced TP–sacral ala distance: 136 of 171 cases (79.5%) had a distance of <5 mm. The reverse relationship was substantially weaker. Of the 427 patients with a TP–sacral ala distance < 5 mm, only 136 (31.9%) fulfilled the morphological criteria for Castellvi Type I. These findings indicate that a short TP–sacral ala distance and transverse process dysplasia are related but non-equivalent anatomical features. Radiographic near-contact (distance of <1 mm) was observed in patients both with and without Castellvi Type I morphology (45/103, 43.7% versus 58/103, 56.3%), indicating that this finding was not restricted to patients meeting the morphological criteria for Castellvi Type I.

Nevertheless, the 10-mm threshold used to define Type I has not yet undergone independent validation against a reference standard such as CT. In the present cohort, it classified approximately 90% of patients (735/817) as having LSTV. While this high detection rate may partly reflect the inclusion of anatomically borderline configurations, the clinical relevance of the 10-mm criterion remains to be established in independent populations.

The prevalence of both a TP–sacral ala distance of <5 mm and true bony contact increased significantly with age, whereas the prevalence of Castellvi Type I remained stable across age groups. The basis for these age-related differences remains uncertain. Age-associated structural changes, including degenerative changes, represent a possible but unverified explanation. However, lumbosacral disc height, osteophyte formation, and other degenerative features were not assessed in the present study; therefore, their potential contribution to the observed pattern cannot be determined.

In patients with Jenkins Type I LSTV, resection of the transverse process yielded significant symptomatic improvement [11], suggesting that Bertolotti syndrome may occur even in the absence of a distinct pseudoarticulation. Furthermore, earlier surgical series and more recent comparative studies suggest that isolated resection of the transverse process or of a true pseudoarticulation may not be the optimal strategy in Type II LSTV; longer-term outcomes appear more favourable with spinal fusion in selected patients [11,12,13].

While the present cohort demonstrated a high prevalence of LSTV, particularly when assessed using the Jenkins classification, LSTV itself was not associated with either documented LBP or the presence of a recorded rheumatologic diagnosis. No statistically significant differences in overall LSTV prevalence were observed between patients with and without a recorded rheumatologic diagnosis, irrespective of the classification system used. Age- and sex-adjusted analyses across all seven prespecified diagnostic categories likewise showed no significant association with Castellvi-positive or Jenkins-positive status after correction for multiple comparisons. Although RA showed a nominal association with Types II–IV, this association did not remain statistically significant after Benjamini–Hochberg correction (raw *p* = 0.009; BH-adjusted *p* = 0.063). The absence of an association between LSTV and LBP also persisted after adjustment for age, sex, radiographic sacroiliitis grade, and the presence of a recorded rheumatologic diagnosis. Within this cohort, these findings do not support an inherent association between transitional lumbosacral anatomy and rheumatologic disorders. Given the specific characteristics of the studied cohort, no definitive assessment of causality between LSTV and LBP was possible. Rheumatologic disease and radiographic sacroiliitis were important potential confounders; although both were accounted for in the multivariable analysis, residual confounding cannot be excluded. The observed increase in LBP prevalence across higher radiographic sacroiliitis grades is consistent with a relationship between greater sacroiliac structural involvement and pain; however, the retrospective data do not permit attribution of LBP to an inflammatory pain mechanism, particularly because Grade 1 changes are equivocal. Moreover, LBP was recorded as a binary variable without identification of a specific pain generator. Consequently, the independent symptomatic relevance of LSTV could not be determined. The presence of LSTV alone does not establish a diagnosis of Bertolotti syndrome. After other causes of LBP have been considered, diagnostic blocks may be used to help determine the clinical relevance of a given morphological variant.

It is noteworthy that the original radiological reports focused primarily on sacroiliac joint findings according to the modified New York criteria and did not mention LSTV. Because these examinations were performed primarily for assessment of the sacroiliac joints, the absence of LSTV reporting should not be interpreted as evidence that the abnormalities were necessarily missed diagnostically. Nevertheless, the findings indicate that the lumbosacral junction may receive limited attention in routine reporting of sacroiliac joint radiographs.

This is of particular clinical relevance for Types II–IV, where the presence of pseudoarticulation or osseous fusion may increase the risk of incorrect vertebral numbering prior to spinal injections or surgery [14,15].

The prevalence of LSTV varies considerably between studies, ranging from 4–35%, with most estimates in the range of 8 to 25% [16,17,18,19]. It is worth noting that many authors do not regard Castellvi Type I as a true transitional anomaly, either excluding it from analyses or noting its limited clinical significance [18,19]. When the analysis is restricted to Types II–IV, the prevalence of 9.4% observed in the present study falls within the ranges reported in the literature. In the present study, the identified prevalence was at the upper end of this range for the Castellvi system, and considerably higher for the Jenkins system. Importantly, the age-related increase in Jenkins-positive findings was confined to Type I variants and paralleled the increasing prevalence of short TP–sacral ala distances, whereas Castellvi Type I prevalence remained stable. This divergence indicates that Jenkins Type I classification was strongly age-dependent in this cohort. This finding may partly explain the markedly higher prevalence generated by the Jenkins classification in older patients and supports further validation of the Type I distance criterion before its application in unselected populations.

LSTV may remain under-recognized, particularly when it is clinically asymptomatic or not the primary focus of imaging. In patients with LBP and LSTV, clinical correlation and, when appropriate, targeted diagnostic procedures such as local anaesthetic blocks may help determine whether the transitional anatomy is clinically relevant [20,21].

Standard radiography of the sacroiliac joints remains the first-line modality in rheumatologic diagnostics and can demonstrate many LSTV morphologies when the lumbosacral junction is adequately included. However, CT continues to be the reference standard owing to its superior spatial resolution and multiplanar capability [22,23].

Further research should focus on validating existing classification criteria and determining whether a more comprehensive approach to morphological assessment of the entire lumbosacral junction is warranted. Such an approach may help improve the anatomical characterization of LSTV and its clinical correlation.

## 5. Limitations

This study was conducted in a population of patients receiving care in a rheumatology outpatient clinic. These patients may be at increased risk of LBP due to comorbid rheumatologic conditions, such as AS, PsA, and spondyloarthropathies associated with inflammatory bowel disease, which may confound the clinical presentation and overlap with the presence of LSTV. Consequently, the independent clinical significance of LSTV cannot be fully assessed in this cohort.

The cohort was predominantly female, which should also be considered when extrapolating the prevalence estimates to populations with different demographic structures.

Because only adults were included, the findings should not be extrapolated to pediatric populations.

Furthermore, assessments limited to sacroiliac joint radiographs do not permit correlation with the morphology of the entire spine or reliable evaluation of degenerative changes, particularly at the lumbosacral disc. They also prevent the identification of additional features of LSTV that may help distinguish lumbarization from sacralization, such as the total number of lumbar vertebrae or sacral segmentation anomalies.

In addition, millimetre-based TP–sacral ala measurements on AP radiographs may be affected by residual variation in beam angulation, patient positioning, pelvic tilt, and lumbosacral lordosis, despite the use of a standardized radiographic protocol.

Interobserver reliability was assessed across all 817 radiographs in the study cohort. Exact subtype agreement was 82.1% for Castellvi and 86.0% for Jenkins, corresponding to unweighted Cohen’s κ values of 0.656 and 0.707, respectively. Binary agreement was higher, reaching 87.1% for Castellvi and 95.3% for Jenkins. Disagreements in exact subtype classification occurred predominantly among Type I variants, particularly in distinctions between negative findings and Type I morphology and between unilateral Type IA and bilateral Type IB configurations. Similar limitations have been reported previously; Farshad-Amacker et al. demonstrated only moderate interreader agreement for LSTV detection and lower agreement for subtype classification on standard AP radiographs [23]. As independent reliability assessment was performed by a single additional observer, the reproducibility of both classifications across readers with different levels of experience and from different specialties remains uncertain. Interobserver reliability was assessed only for the final classification (Castellvi and Jenkins subtypes). The TP–sacral ala distances were not independently remeasured; therefore, no formal evaluation of measurement reliability, such as an intraclass correlation coefficient or Bland–Altman analysis, could be performed. Consequently, the reproducibility of the millimetre-based thresholds used in the Jenkins classification and in the sensitivity analyses remains unquantified.

Radiographs were acquired using two digital radiography systems, with beam angulation and patient positioning adjusted as necessary to optimize visualization of the sacroiliac joints. Although minor variations in projection geometry cannot be entirely excluded, all examinations were performed according to a standardized institutional protocol. Nevertheless, pelvic orientation and lumbosacral alignment may contribute to some degree of measurement variability. As these parameters were not directly quantified, their potential influence could not be assessed separately. This consideration is particularly relevant to age-related analyses, given that lumbosacral anatomy and pelvic alignment may vary with age, although the extent to which such variations may have affected the present measurements remains uncertain.

Finally, LBP was retrospectively recorded as a binary clinical variable without consistent information regarding duration, mechanical versus inflammatory characteristics, laterality, or attribution to transitional lumbosacral anatomy. This limits conclusions regarding the clinical significance of LSTV and precludes assessment of Bertolotti syndrome itself.

## 6. Conclusions

Application of the Castellvi and Jenkins classification systems to the same rheumatology cohort yielded markedly different prevalence estimates of LSTV (30.4% vs. 90.0%, respectively). This discrepancy was attributable predominantly to Type I variants, whereas both systems identified the same 77 patients (9.4%) as having Types II–IV. These findings indicate that the distinct diagnostic criteria employed by the two classification systems, particularly the distance-based criterion defining Jenkins Type I, substantially influence the resulting prevalence estimates. Although the present study does not establish the diagnostic accuracy or clinical utility of either system relative to the other, the marked age-related increase in the prevalence of Jenkins Type I, in contrast to the absence of a significant age-related association with LSTV prevalence under the Castellvi classification, highlights the need for further research to clarify the influence of differing classification criteria on prevalence estimates and to independently validate these criteria.

Sacroiliac joint radiographs provide an opportunity to assess the lumbosacral junction during routine imaging. Among examinations meeting the predefined imaging-coverage criteria, the lumbosacral junction was assessable, yet LSTV was not mentioned in any of the corresponding radiology reports. These findings suggest that systematic assessment of the lumbosacral junction may improve the recognition and reporting of LSTV in patients undergoing sacroiliac joint radiography.

## Figures and Tables

**Table 1:** Age-stratified prevalence of LSTV according to the Castellvi and Jenkins classification systems. Cochran–Armitage trend test: Jenkins, Z = 6.80, *p* < 0.001; Castellvi, Z = −0.09, *p* = 0.929.

Age Group (Years)	*n*	Jenkins-Positive, *n* (%)	Castellvi-Positive, *n* (%)
<20	19	13 (68.4%)	7 (36.8%)
20–29	80	59 (73.8%)	23 (28.8%)
30–39	132	113 (85.6%)	39 (29.5%)
40–49	205	186 (90.7%)	61 (29.8%)
50–59	188	175 (93.1%)	60 (31.9%)
60–69	125	121 (96.8%)	41 (32.8%)
70–79	62	62 (100.0%)	15 (24.2%)
80–89	6	6 (100.0%)	2 (33.3%)
Total	817	735 (90.0%)	248 (30.4%)

**Table 2:** Disease-specific associations between rheumatologic diagnoses and LSTV.

**Panel A. Castellvi-positive status**					
**Diagnosis**	***N***	**Castellvi-positive, *n* (%)**	**aOR (95% CI)**	**Raw *p***	**BH-adjusted *p***
No recorded rheumatologic diagnosis	367	108 (29.4)	Reference	—	—
Ankylosing spondylitis (AS)	128	42 (32.8)	1.07 (0.69–1.67)	0.768	0.768
Psoriatic arthritis (PsA)	116	29 (25.0)	0.80 (0.49–1.31)	0.383	0.614
Degenerative joint disease	61	22 (36.1)	1.38 (0.75–2.55)	0.304	0.614
Non-radiographic axial spondyloarthritis (nr-axSpA)	52	15 (28.8)	0.90 (0.47–1.75)	0.761	0.768
Rheumatoid arthritis (RA)	52	18 (34.6)	1.65 (0.86–3.15)	0.131	0.614
Systemic lupus erythematosus (SLE)	13	2 (15.4)	0.54 (0.11–2.56)	0.437	0.614
Undifferentiated connective tissue disease (UCTD)	10	2 (20.0)	0.53 (0.11–2.64)	0.439	0.614
**Panel B. Jenkins-positive status**					
**Diagnosis**	***N***	**Jenkins-positive, *n* (%)**	**aOR (95% CI)**	**Raw *p***	**BH-adjusted *p***
No recorded rheumatologic diagnosis	367	331 (90.2)	Reference	—	—
Ankylosing spondylitis (AS)	128	116 (90.6)	1.02 (0.50–2.07)	0.961	0.961
Psoriatic arthritis (PsA)	116	103 (88.8)	0.70 (0.35–1.41)	0.317	0.610
Degenerative joint disease	61	58 (95.1)	0.91 (0.25–3.32)	0.884	0.961
Non-radiographic axial spondyloarthritis (nr-axSpA)	52	45 (86.5)	0.49 (0.20–1.22)	0.127	0.541
Rheumatoid arthritis (RA)	52	50 (96.2)	2.04 (0.46–9.13)	0.349	0.610
Systemic lupus erythematosus (SLE)	13	11 (84.6)	0.68 (0.13–3.57)	0.650	0.910
Undifferentiated connective tissue disease (UCTD)	10	8 (80.0)	0.30 (0.06–1.57)	0.154	0.541
**Panel C. Types II–IV**					
**Diagnosis**	***N***	**Types II–IV, *n* (%)**	**aOR (95% CI)**	**Raw *p***	**BH-adjusted *p***
No recorded rheumatologic diagnosis	367	28 (7.6)	Reference	—	—
Ankylosing spondylitis (AS)	128	11 (8.6)	1.12 (0.54–2.33)	0.767	0.894
Psoriatic arthritis (PsA)	116	11 (9.5)	1.29 (0.62–2.70)	0.495	0.859
Degenerative joint disease	61	8 (13.1)	1.71 (0.69–4.19)	0.244	0.829
Non-radiographic axial spondyloarthritis (nr-axSpA)	52	3 (5.8)	0.73 (0.21–2.51)	0.613	0.859
Rheumatoid arthritis (RA)	52	10 (19.2)	3.01 (1.32–6.90)	0.009	0.063
Systemic lupus erythematosus (SLE)	13	1 (7.7)	1.02 (0.13–8.18)	0.987	0.987
Undifferentiated connective tissue disease (UCTD)	10	0 (0.0)	NE	0.355 *	0.829

Values are presented as *n* (%). aORs were adjusted for age and sex, with patients without a recorded rheumatologic diagnosis as the reference group. BH-adjusted *p* values were calculated separately within each outcome across the seven prespecified diagnostic categories. NE, not estimable because of complete separation (no Types II–IV cases in the UCTD group). * Age- and sex-adjusted score-test p value due to complete separation.

## Data Availability

The de-identified data supporting the findings of this study are available from the corresponding author upon reasonable request, subject to ethical and privacy restrictions.

## Supplementary Materials

The following supporting information can be downloaded at: https://www.mdpi.com/article/10.3390/jcm15218436/s1, Table S1: Side-specific sacroiliac joint grades according to the modified New York criteria and coexisting LSTV findings; Table S2: Cross-tabulation of LSTV subtypes according to the Castellvi and Jenkins classification systems in the study cohort.

## Abbreviations

AEC	automatic exposure control;
AI	artificial intelligence;
AP	anteroposterior;
AS	ankylosing spondylitis;
DISH	diffuse idiopathic skeletal hyperostosis;
LBP	low back pain;
LSTV	lumbosacral transitional vertebrae;
nr-axSpA	non-radiographic axial spondyloarthritis;
PACS	Picture Archiving and Communication System;
PsA	psoriatic arthritis;
RA	rheumatoid arthritis;
SLE	systemic lupus erythematosus;
TP	transverse process;
UCTD	undifferentiated connective tissue disease;
YLD	years lived with disability.

## References

[1] (2016). GBD 2015 Disease and Injury Incidence and Prevalence Collaborators. Global, regional, and national incidence, prevalence, and years lived with disability for 310 diseases and injuries, 1990–2015: A systematic analysis for the Global Burden of Disease Study 2015. Lancet.

[2] Hartvigsen J., Hancock M.J., Kongsted A., Quinette L., Manuela L.F., Stephane G., Damian H., Jaro K., Glenn P., Joachim S. (2018). What low back pain is and why we need to pay attention. Lancet.

[3] McGrath K.A., Rabah N.M., Steinmetz M.P. (2021). Identifying treatment patterns in patients with Bertolotti syndrome: An elusive cause of chronic low back pain. Spine J..

[4] Castellvi A.E., Goldstein L.A., Chan D.P. (1984). Lumbosacral transitional vertebrae and their relationship with lumbar extradural defects. Spine.

[5] Bertolotti M. (1917). Contributo alla conoscenza dei vizi di differenziazione regionale del rachide con speciale riguardo all assimilazione sacrale della v. lombare. Radiol. Med..

[6] Quinlan J.F., Duke D., Eustace S. (2006). Bertolotti’s syndrome. A cause of back pain in young people. J. Bone Jt. Surg. Br..

[7] Southworth J.D., Bersack S.R. (1950). Anomalies of the lumbosacral vertebrae in 550 individuals without symptoms referable to the low back. Am. J. Roentgenol. Radium Ther..

[8] Jenkins A.L., O’Donnell J., Chung R.J., Jenkins S., Hawks C., Lazarus D., McCaffrey T., Terai H., Harvie C. (2023). Redefining the Classification for Bertolotti Syndrome: Anatomical Findings in Lumbosacral Transitional Vertebrae Guide Treatment Selection. World Neurosurg..

[9] Krenn V.A., Fornai C., Webb N.M., Woodert M.A., Prosch H., Haeusler M. (2022). The morphological consequences of segmentation anomalies in the human sacrum. Am. J. Biol. Anthropol..

[10] Wu B., Xiao B., Wu M., Wang L., Rong T., Zhang D., Duan S., Liu B. (2026). A rapid method to identify lumbarization on lumbar spine radiography and computed tomography. Eur. Spine J..

[11] Jenkins A.L., Chung R.J., O’Donnell J., Hawks C., Jenkins S., Lazarus D., McCaffrey T., Terai H., Harvie C., Matsuokas S. (2023). Redefining the Treatment of Lumbosacral Transitional Vertebrae for Bertolotti Syndrome: Long-Term Outcomes Utilizing the Jenkins Classification to Determine Treatment. World Neurosurg..

[12] Santavirta S., Tallroth K., Ylinen P., Suoranta H. (1993). Surgical treatment of Bertolotti’s syndrome. Follow-up of 16 patients. Arch. Orthop. Trauma Surg..

[13] Mikula A.L., Lakomkin N., Ransom R.C., Flanigan P.M., Waksdahl L.A., Pennington Z., Sharma M.S., Elder B.D., Fogelson J.L. (2022). Operative Treatment of Bertolotti Syndrome: Resection Versus Fusion. World Neurosurg..

[14] French H.D., Somasundaram A.J., Schaefer N.R., Laherty R.W. (2014). Lumbosacral transitional vertebrae and its prevalence in the Australian population. Glob. Spine J..

[15] Konin G.P., Walz D.M. (2010). Lumbosacral transitional vertebrae: Classification, imaging findings, and clinical relevance. AJNR Am. J. Neuroradiol..

[16] Fang H., Wang J., Kiwale J.C., Cui M., Zeng X., Yang C., Zhang Y., Xie L. (2025). Prevalence, diagnosis, and clinical significance of lumbosacral transitional vertebrae: A systematic review and narrative analysis. Brain Spine.

[17] Nardo L., Alizai H., Virayavanich W., Liu F., Hernandez A., Lynch J.A., Nevit M.C., McCulloch C.E., Lane N.E., Link T.M. (2012). Lumbosacral transitional vertebrae: Association with low back pain. Radiology.

[18] Vinha A., Bártolo J., Lemos C., Cordeiro F., Rodrigues-Pinto R. (2022). Lumbosacral transitional vertebrae: Prevalence in a southern European population and its association with low back pain. Eur. Spine J..

[19] Marks R.C., Thulbourne T. (1991). Infiltration of anomalous lumbosacral articulations. Steroid and anesthetic injections in 10 back-pain patients. Acta Orthop. Scand..

[20] Glémarec J., Varin S., Cozic C., Tanguy G., Volteau C., Montigny P., Le Goff B., Lafitte C.D., Maugars Y. (2018). Efficacy of local glucocorticoid after local anesthetic in low back pain with lumbosacral transitional vertebra: A randomized placebo-controlled double-blind trial. Jt. Bone Spine.

[21] Hanhivaara J., Määttä J.H., Kinnunen P., Niinimäki J., Nevalainen M.T. (2024). Castellvi classification of lumbosacral transitional vertebrae: Comparison between conventional radiography, CT, and MRI. Acta Radiol..

[22] Andrade-Andrade P., Acevedo-González J.C. (2026). Radiologic Insights: Diagnosing Lumbosacral Transitional Vertebrae. Systematic Review of the Literature. Pain Pract..

[23] Farshad-Amacker N.A., Lurie B., Herzog R.J., Hughes A.P., Aichmair A., Farshad M. (2014). Interreader and intermodality reliability of standard anteroposterior radiograph and magnetic resonance imaging in detection and classification of lumbosacral transitional vertebra. Spine J..

