# Supplementary material for: Lumbosacral Transitional Vertebrae Detected on Routine Sacroiliac Joint Radiographs in a Rheumatology Cohort: Comparison of Castellvi and Jenkins Systems
Source: J Clin Med. 2026 Oct 29;15(21):8436. doi: 10.3390/jcm15218436 (PMC13764135; doi:10.3390/jcm15218436)
Supplement: Supplementary file 1 [file jcm-15-08436-s001.zip › Table S1.pdf]

Supplementary Table S1. Side-specific distribution of radiographic sacroiliitis grades according to the modified New York criteria, with coexisting LSTV findings according to the Castellvi and Jenkins classifications. *Single values indicate equal grades in both sacroiliac joints, whereas paired values indicate the right- and left-sided grades, respectively. The Castellvi-positive and Jenkins-positive columns are not mutually exclusive because the same patient may be classified as positive by both systems. The final column includes patients classified as negative according to both the Castellvi and Jenkins systems. Configurations not observed in the study cohort were omitted.*

SIJ grade configuration (R/L)	N	Castellvi-positive, n	Jenkins-positive, n	Negative according to both systems, n
0	210	72	188	22
0/1	35	6	34	1
0/2	19	8	18	1
0/3	1	0	0	1
1	217	62	197	19
1/0	61	16	53	8
1/2	19	4	15	4
1/3	4	3	4	0
2	127	36	116	10
2/0	34	8	29	5
2/1	29	13	26	3
2/3	12	5	12	0
3	26	9	23	3
3/0	4	0	3	1
3/1	1	1	1	0
3/2	5	1	4	1
3/4	1	0	1	0
4	9	3	8	1
4/3	3	1	3	0
**Total**	**817**	**248**	**735**	**80**
