# Supplementary material for: Lumbosacral Transitional Vertebrae Detected on Routine Sacroiliac Joint Radiographs in a Rheumatology Cohort: Comparison of Castellvi and Jenkins Systems
Source: J Clin Med. 2026 Oct 29;15(21):8436. doi: 10.3390/jcm15218436 (PMC13764135; doi:10.3390/jcm15218436)
Supplement: Supplementary file 1 [file jcm-15-08436-s001.zip › Table S2.pdf]

Supplementary Table S2. Cross-tabulation of LSTV subtypes according to the Castellvi and Jenkins classification systems in the study cohort (n = 817). Values represent numbers of patients.

Castellvi ↓ / Jenkins →	Negative	IA	IB	IIA	IIB	IIC	III	IVA	IVB	IVC	**Total**
Negative	80	69	420	0	0	0	0	0	0	0	**569**
IA	0	11	83	0	0	0	0	0	0	0	**94**
IB	2	3	72	0	0	0	0	0	0	0	**77**
IIA	0	0	0	9	0	20	0	0	0	0	**29**
IIB	0	0	0	0	17	0	0	0	0	0	**17**
IIIA	0	0	0	0	0	0	0	6	0	6	**12**
IIIB	0	0	0	0	0	0	12	0	0	0	**12**
IV	0	0	0	0	0	0	0	0	7	0	**7**
**Total**	**82**	**83**	**575**	**9**	**17**	**20**	**12**	**6**	**7**	**6**	**817**
